# Health Tract, No. 105

**Published:** 1862-09

**Authors:** 


HEALTH TRACT, No. 105.

### MARRIAGE.

Marriage is the natural state of human kind. There never can be lasting good health without it; it is an impossibility, except combined with criminal practices. A person may live in good health to the age of twenty-five, but if marriage is deferred beyond that, every month's delay is the eating out, more and more, the very essence of life, and the worm of certain disease and premature death burrows the more deeply into the vitals. On the other hand, marriage not later than twenty-five, prolongs life. It was for this reason, noticed some three thousand years ago, that the ancients dedicated a temple to Hymen, the god of youth; that is, "to the deity which prolongs youth." Men and women get older more rapidly when they remain single, and die off more rapidly; the men from falling into dissipated habits and irregularities. The woman, true to nature's instincts, and living in her purity, grows less and less vivacious, and by slow degrees settles down in inaction, in feebleness, and premature decline.

As long as a man is unmarried, he feels himself unfixed, unsettled; and keen business men consider him insecure, because he can any day pack up his trunk and disappear. The most magnificent swindlers in Wall street, those for the very largest amounts, were unmarried men.

There has always existed, from very early ages, a general and almost instinctive prejudice against those who remain unmarried after thirty. Lycurgus legislated against celibacy, and Cato outlawed female celibates at twenty-five, and bachelors of thirty-five. It was a creed of the earlier nations, that the souls of those who died unmarried, were doomed to eternal wanderings.

In the present state of society, if the daughter should be encouraged to marry at twenty-one, and the son at twenty-five, vigorous health and moral purity would be promoted thereby. Pride and cowardice join in delaying marriage; but let the fearful statistics of the larger cities of the world tell the sad story of demoralization. In Milan there are thirty-two illegitimates out of every hundred children born; in Paris thirty-three, in Brussels thirty-five, in Munich forty-eight, in Vienna fifty-one.

Out of every hundred suicides, sixty-seven are single, thirty-three married.

Of the hapless insane, out of one hundred and seventy-two, ninety-eight were single, seventy-four married.

Celibacy is a constant cause of premature death. Of one hundred and twenty who are forty-eight years old, eighty will be married, only forty single. In one hundred single men, only twenty-two will live to be sixty years old. Of one hundred married men, forty-eight will live to that age. Of a dozen men of eighty years, nine will be married, three single. Not only marry young, but marry out of your family. The effects of marrying cousins, for example, even to the third degree, are fearful to contemplate. Of one hundred and fifty-four cousin-marriages, in Dublin, there were one hundred deaf and dumb children. Dr. Buxton, of Liverpool, states, that in one hundred and nine such marriages, each family had one deaf and dumb child; thirty-eight of them had two deaf mutes; in seventeen there were three; three had four; one had six; one had seven, and one had eight deaf mutes — that is, two hundred and sixty-nine children born deaf and dumb, to one one hundred and nine cousin-marriages. The consanguineous marriages in France are two per cent of the whole population. Of their children, twenty-eight per cent are deaf mutes in Paris, twenty-five at Lyons, thirty at Bourdeaux; while as to the Jews, twenty-seven per cent of the offspring of such marriages are deaf mutes, one sixth per cent of Christian parents; Jews oftener marrying blood relations.

In England, where Bible teachings more than in any other country prevail, and discountenance consanguineous marriages, as well as private profligacy, only six per cent of such children born are deaf mutes, instead of thirty, as when the English do marry relations, they are more distant; and only six per cent of those born are illegitimate, instead of fifty-one per cent, as the direct result of the teachings of that blessed book.

